# Progression patterns and therapeutic sequencing following immune checkpoint inhibition for hepatocellular carcinoma: An international observational study

**DOI:** 10.1111/liv.15502

**Published:** 2023-01-13

**Authors:** Thomas Talbot, Antonio D'Alessio, Matthias Pinter, Lorenz Balcar, Bernhard Scheiner, Thomas U. Marron, Tomi Jun, Sirish Dharmapuri, Celina Ang, Anwaar Saeed, Hannah Hildebrand, Mahvish Muzaffar, Claudia A. M. Fulgenzi, Suneetha Amara, Abdul Rafeh Naqash, Anuhya Gampa, Anjana Pillai, Yinghong Wang, Uqba Khan, Pei‐Chang Lee, Yi‐Hsiang Huang, Bertram Bengsch, Dominik Bettinger, Yehia I. Mohamed, Ahmed Kaseb, Tiziana Pressiani, Nicola Personeni, Lorenza Rimassa, Naoshi Nishida, Masatoshi Kudo, Arndt Weinmann, Peter R. Galle, Ambreen Muhammed, Alessio Cortellini, Arndt Vogel, David J. Pinato

**Affiliations:** ^1^ Department of Surgery & Cancer, Imperial College London Hammersmith Hospital London UK; ^2^ Department of Biomedical Sciences Humanitas University Milan Italy; ^3^ Division of Gastroenterology & Hepatology, Department of Internal Medicine III Medical University of Vienna Vienna Austria; ^4^ Division of Hematology/Oncology, Department of Medicine, Tisch Cancer Institute Mount Sinai Hospital New York New York USA; ^5^ Sema4 Stamford Connecticut USA; ^6^ Division of Medical Oncology, Department of Medicine Kansas University Cancer Center Westwood Kansas USA; ^7^ Division of Hematology/Oncology East Carolina University Greenville North Carolina USA; ^8^ Department of Medical Oncology University Campus Bio‐Medico Rome Italy; ^9^ Division of Cancer Treatment and Diagnosis National Cancer Institute Bethesda Maryland USA; ^10^ Section of Gastroenterology Hepatology & Nutrition, the University of Chicago Medicine Chicago Illinois USA; ^11^ Department of Gastroenterology, Hepatology & Nutrition The University of Texas MD Anderson Cancer Center Houston Texas USA; ^12^ Division of Hematology and Oncology Weill Cornell Medicine/New York Presbyterian Hospital New York New York USA; ^13^ Division of Gastroenterology and Hepatology, Department of Medicine Taipei Veterans General Hospital Taipei Taiwan; ^14^ Institute of Clinical Medicine National Yang Ming Chiao Tung University School of Medicine Taipei Taiwan; ^15^ Department of Medicine II, Faculty of Medicine Medical Center University of Freiburg, University of Freiburg Freiburg Germany; ^16^ Department of Gastrointestinal Medical Oncology The University of Texas MD Anderson Cancer Center Houston Texas USA; ^17^ Medical Oncology and Hematology Unit, Humanitas Cancer Center IRCCS Humanitas Research Hospital Milan Italy; ^18^ Department of Gastroenterology and Hepatology Kindai University Faculty of Medicine Osaka‐Sayama Japan; ^19^ 1st Department of Internal Medicine, Gastroenterology and Hepatology University Medical Center of the Johannes Gutenberg‐University Mainz Mainz Germany; ^20^ Department of Gastroenterology, Hepatology and Endocrinology Hannover Medical School Hannover Germany; ^21^ Division of Oncology, Department of Translational Medicine University of Piemonte Orientale “A. Avogadro” Novara Italy

## Abstract

**Background and Aims:**

Different approaches are available after the progression of disease (PD) to immune checkpoint inhibitors (ICIs) for hepatocellular carcinoma (HCC), including the continuation of ICI, treatment switching to tyrosine kinase inhibitors (TKIs) and cessation of anticancer therapy. We sought to characterise the relationship between radiological patterns of progression and survival post‐ICI, also appraising treatment strategies.

**Methods:**

We screened 604 HCC patients treated with ICIs, including only those who experienced PD by data cut‐off. We evaluated post‐progression survival (PPS) according to the treatment strategy at PD and verified its relationship with radiological patterns of progression: intrahepatic growth (IHG), new intrahepatic lesion (NIH), extrahepatic growth (EHG), new extrahepatic lesion (NEH) and new vascular invasion (nVI).

**Results:**

Of 604 patients, 364 (60.3%) experienced PD during observation. Median PPS was 5.3 months (95% CI: 4.4–6.9; 271 events). At the data cut‐off, 165 patients (45%) received no post‐progression anticancer therapy; 64 patients (17.6%) continued ICI beyond PD. IHG (HR 1.64 [95% CI: 1.21–2.22]; *p* = .0013) and nVI (HR 2.15 [95% CI: 1.38–3.35]; *p* = .0007) were associated with shorter PPS. Multivariate models adjusted for progression patterns, treatment line and albumin‐bilirubin grade and Eastern Cooperative Oncology Group performance status at PD confirmed receipt of ICI beyond PD with (HR 0.17, 95% CI: 0.09–0.32; *p* < .0001) or without subsequent TKI (HR 0.39, 95% CI: 0.26–0.58; *p* < .0001) as predictors of prolonged PPS versus no anticancer therapy.

**Conclusions:**

ICI‐TKI sequencing is a consolidated option in advanced HCC. nVI and IHG predict a poorer prognosis. Despite lack of recommendation, the continuation of ICI beyond progression in HCC is adopted clinically: future efforts should appraise which patients benefit from this approach.

**Abbreviations**
ACT: anticancer therapy
ALBI: albumin‐bilirubin
BCLC: Barcelona Clinic Liver Cancer
CI: confidence interval
CTLA‐4: cytotoxic T‐lymphocyte‐associated protein‐4
ECOG‐PS: Eastern Cooperative Oncology Group performance status
EHG: extrahepatic growth
HCC: hepatocellular carcinoma
HR: hazard ratio
ICI: immune checkpoint inhibitor
IHG: intrahepatic growth
LRT: loco‐regional therapy
NEH: new extrahepatic lesion(s)
NIH: new intrahepatic lesion(s)
NSCLC: non‐small‐cell lung cancer
nVI: new vascular invasion
OS: overall survival
PD: progressive disease
PD‐1: programmed cell death‐1
PD‐L1: programmed cell death ligand‐1
PFS: progression‐free survival
PPS: post‐progression survival
RECIST: response evaluation criteria in solid tumours
TACE: trans‐arterial chemoembolization
TKI: tyrosine kinase inhibitor
VEGF: vascular endothelial growth factor

**Key points**

- Of the 364 studied patients, the median post‐progression survival (PPS) after progressive disease on immune checkpoint inhibitors was 5.3 months.
- 45% of patients received no further anticancer therapy after disease progression.
- Continuation of immunotherapy after documented hepatocellular carcinoma (HCC) disease progression has been adopted in clinical practice, with 17.6% of patients in our cohort continuing treatment.
- In contrast to sorafenib, intra‐hepatic HCC growth and new vascular invasion are associated with poorer outcomes after progression on immunotherapy.
- Both continuation of immunotherapy and treatment switching to tyrosine kinase inhibitors were associated with improved PPS.

## 1 INTRODUCTION

Advanced hepatocellular carcinoma (HCC) has historically carried a poor prognosis in view of limited treatment options. Sorafenib, a tyrosine kinase inhibitor (TKI), represented the first breakthrough in the treatment of advanced HCC, increasing median overall survival (OS) compared to placebo from 7.9 to 10.7 months^1^ and remained, for over a decade, the only systemic therapy available to patients with HCC. More recently, immune checkpoint inhibitors (ICIs) have gained significant traction in the treatment of HCC. While HCC is moderately responsive to programmed cell death‐1 inhibitors, the combination of atezolizumab plus bevacizumab yielded a median OS of 19.2 months compared to 13.4 observed with sorafenib,^2^, ^3^ paving the way for combination immunotherapy in the treatment of HCC. While the dual programmed cell death ligand‐1 (PD‐L1)/vascular endothelial growth factor inhibitor combination has yielded undoubted superiority over sorafenib, not all patients are candidates for combination immunotherapy owing to concerns over toxicity.

Other immunotherapeutic approaches are available to sorafenib‐experienced patients, such as pembrolizumab and nivolumab plus ipilimumab combination therapy (as well as previously nivolumab monotherapy), though survival benefit has been called into question, and access to these therapies is restricted to only certain healthcare systems.^4^ While the higher quality of evidence is available for molecularly targeted therapies following progression of disease (PD) on sorafenib,^5^, ^6^ there is no randomised trial evidence to suggest whether monotherapy or combination immunotherapy followed by second‐line TKI leads to superior long‐term survival outcomes compared to initial TKI use followed by either subsequent TKI or immunotherapy in the second line.

A key issue that complicates the optimal sequencing of systemic therapy is the heterogeneity of advanced HCC, where tumour progression and often contemporaneous progressive liver dysfunction dictate survival. As a result of this complex and dynamic interplay, there is high attrition from first‐ to second‐line therapy in advanced disease: over 50% of patients who progress on first‐line TKI are no longer eligible to further systemic therapy.^7^, ^8^ In patients who remain eligible for second‐line therapy post‐TKI, radiological distribution of disease at the point of progression has been identified as a strong determinant of survival, leading to the proposal of a Barcelona Clinic Liver Cancer (BCLC) subclassification of advanced HCC that incorporates patterns of radiological progression to optimise patient selection for further therapy. Development of new extrahepatic disease and new vascular invasion (nVI) significantly predict for poorer post‐progression survival (PPS) following PD on sorafenib.^7^ However, the relationship between radiological patterns of progression and ICI therapy has not been established.

Different anticancer strategies are available to patients who experience PD on ICI, including treatment switching to TKIs.^9^, ^10^ Unlike sorafenib, a proportion of patients on ICI therapy can achieve significant disease downstaging, leading to the effective utilisation of loco‐regional or operative therapies in selected patients.^3^, ^4^ Additionally, it is becoming increasingly recognised that clinical benefit may be registered in patients who continue to receive immunotherapy beyond radiological PD, a practice that is consolidated in selected patients with non‐small‐cell lung cancer and melanoma; especially in the case of oligo‐progressive disease.^11^, ^12^

Given the increasingly prevalent use of ICIs in the treatment of HCC, we designed this study with two aims. First, we intended to characterise the clinical features and radiological patterns of disease progression post‐ICI and evaluate their relationship with clinical outcomes. Secondly, we aimed to retrospectively describe how different therapeutic sequencing strategies are associated with clinical outcomes in patients who, at the point of first radiological progression from ICI, remain eligible for further anticancer therapy.

## 2 METHODS

### 2.1 Study population

From an international consortium of 13 tertiary‐care referral centres located in Europe, the United States and Asia, we accrued a prospectively maintained cohort of HCC patients undergoing treatment with ICIs between 2017 and 2021 (Table S1). Demographic and clinical data were collected retrospectively and curated at each participating centre. Eligible patients were required to fulfil the following inclusion criteria: (1) diagnosis of HCC by histopathological confirmation or imaging criteria according to the American Association for the study of Liver Disease^13^ and European Association for the Study of the Liver^14^ guidelines, (2) eligible for ICI monotherapy or combination therapy for HCC, not amenable to curative or loco‐regional therapy (LRT) following local multidisciplinary tumour board review and (3) have measurable disease according to response evaluation criteria in solid tumours (RECIST) 1.1 criteria^15^ at ICI commencement. At the censoring date of 30 April 2021, the multi‐centre database included 604 eligible patients.

Ethical approval to conduct this study was granted by the Imperial College Tissue Bank (Reference Number R16008). The Institutional Review Board in each participating institution approved the study protocol. All study‐related procedures and data collection were conducted in accordance with the World Medical Association Declaration of Helsinki and the National Institute for Health Research Good Clinical Practice. Participants gave written informed consent for inclusion in the study.

### 2.2 Study end points

This analysis aimed to determine the clinical characteristics of HCC patients treated with ICI and evaluate the clinical outcomes of these patients following PD. Patients were evaluated with re‐staging imaging every 8–12 weeks by the treating physician according to RECIST v1.1 criteria. After screening all the entered patients, we selected only those with documented PD at data cut‐off on the basis of RECIST v1.1 criteria as determined by local investigator review. For each of these patients, we calculated PPS defined as the time between the documented first occurrence of PD on ICI therapy until death or the last follow‐up (for censored patients). Patients alive at the data cut‐off were censored at the date of the last clinical follow‐up.

We firstly reported the clinical characteristics of patients described at baseline (i.e. prior to immunotherapy) and evaluated these according to the receipt of any post‐progression anticancer therapy. In addition, we evaluated whether receipt of active treatment post‐progression was associated with disease characteristics of validated prognostic significance in advanced HCC, including Eastern Cooperative Oncology Group performance status (ECOG‐PS) at PD, Albumin‐Bilirubin (ALBI) grade at PD and change in ALBI grade between ICI commencement and PD (categorised as deteriorated, unchanged or improved). We also assessed whether receipt of active treatment after disease progression was associated with clinical benefit from the previous line of treatment, measured through median progression‐free survival (PFS—computed from the beginning of the ICI therapy to the first occurrence of disease progression).

We then evaluated the relationship between PPS according to the treatment strategy at disease progression as follows: no post‐progression anticancer treatment, ICI therapy beyond disease progression (ICI beyond PD) without subsequent TKI, post‐progression TKI without continuation of ICI beyond PD, ICI beyond PD and subsequent TKI and other anticancer therapies (including systemic chemotherapy, LRTs and investigational agents within clinical trials). TKIs included sorafenib, regorafenib, lenvatinib and cabozantinib. As a receptor tyrosine kinase‐targeting agent, ramucirumab was also included in this group. Post‐progression continuation of ICI was defined as the continuation of treatment for more than 15 days after documented radiological PD. Switching to a different ICI (with or without a combination agent) at progression was not classed as the continuation of ICI beyond PD.

We reported the radiological patterns of disease progression and documented their distribution according to post‐progression treatment strategies. Radiological patterns of progression were categorised as described previously by Reig et al.^7^: intrahepatic growth (IHG), new intrahepatic lesion(s) (NIH), extrahepatic growth (EHG), new extrahepatic lesion(s) (NEH) and nVI. We separately evaluated associations between each treatment strategy and radiological patterns of progression, ECOG‐PS and ALBI grade at disease progression, ALBI grade variation and treatment line of ICI‐based therapy. The impact of each pattern of progression on PPS was evaluated separately in univariable analyses. Acknowledging that different patterns of progression can present at the same time, we described radiological pattern overlaps and then developed a fixed multivariable regression model including post‐progression treatment strategies, all radiological patterns of progression, ECOG‐PS and ALBI grade at disease progression and ICI treatment line. In addition, we performed an explorative analysis including only patients who experienced non‐overlapped patterns of disease progression, which were considered as a unique categorical variable.

Lastly, we performed an ancillary analysis of post‐progression therapeutic strategies among patients who received ICI‐based therapy as first‐line systemic treatment separately.

### 2.3 Statistical analysis

Demographic data were summarised using descriptive statistics. Median PPS was calculated using the Kaplan–Meier survival method and the median period of follow‐up was calculated according to the reverse Kaplan–Meier method. Log‐rank test was used for the univariable analysis of PPS. The chi‐square test was used to compare categorical variables. Given the lack of censored data, the Mann–Whitney test was used to compare the time distribution for PFS of patients receiving and patients not receiving post‐progression therapy. Cox proportional hazards regression was used for the univariable analysis of PPS according to the radiological patterns of disease progression, the multivariable analysis of PPS and to compute the hazard ratios (HRs) for death with 95% confidence intervals (CIs). PPS curve plots were generated using the Kaplan–Meier method. Cox regression survival probability plots for PPS were generated according to the presence of a given radiological pattern of progression. Each curve was obtained from separate multivariable models and superimposed, incorporating ECOG‐PS at disease progression (0 and 1 vs. ≥2), ICI treatment line (1st vs. non‐1st), ICI beyond PD and post‐progression TKI exposure as adjusting factors.

The alpha level for all analyses was set to *p* < .05. All statistical analyses were performed using the MedCalc Statistical Software version 19.3.1 (MedCalc Software Ltd, Ostend, Belgium; https://www.medcalc.org; 2020).

## 3 RESULTS

### 3.1 Patient characteristics at immune checkpoint inhibitor initiation and at disease progression post‐immune checkpoint inhibitor

**FIGURE 1:**
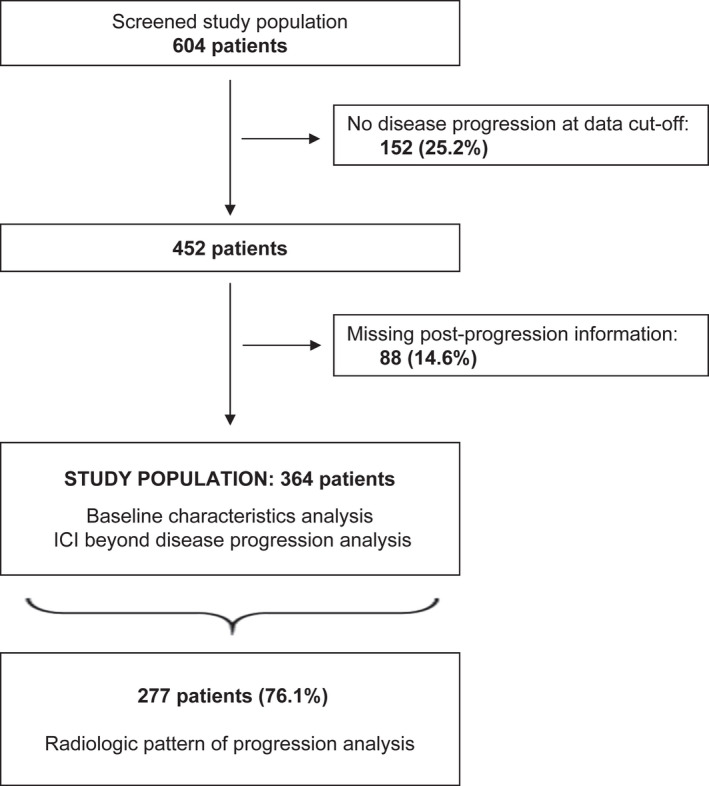
Study consort diagram.

At the data cut‐off, 604 patients were entered into the study, with a median period of follow‐up from ICI initiation of 30.1 months (95% CI: 28.5–32.8). After the exclusion of 152 patients (25.2%) who did not experience disease progression at the data cut‐off and 88 patients (14.6%) with missing information about post‐progression outcomes, the final study population consisted of 364 patients (60.2%). Of these 364 patients, 271 had died at data cut‐off. The median OS of these 271 patients from the initial diagnosis of HCC was 32.7 months (IQR 17.1–56.8). The date of commencement of first‐line systemic therapy was not available. Detailed information about radiological pattern of disease progression was available for 277 patients. A consort flow diagram is available in Figure 1.

Among the 364 included patients, causes of ICI discontinuation included disease progression (276, 75.8%), death (24, 6.5%), toxicity (20, 5.4%) and clinical deterioration (18, 4.9%) with some causes overlapping. Of these 364 patients, 165 (45.3%) did not receive any post‐progression anticancer therapy (including ICI beyond disease progression) at data cut‐off; among them, 132 patients (80%) died. Causes for discontinuation in the 165 patients who did not receive any post‐progression anticancer treatment included disease progression (105, 63.6%), death (16, 9.6%), toxicity (14, 8.4%) and clinical deterioration (16, 9.6%).

Overall, ICI therapy was continued beyond PD in 64 of 364 patients (17.6%). Of these, 16 patients had received combination therapy rather than ICI monotherapy and continued the treatment combination past PD. Of the 64 patients who received ICI beyond PD, 44 patients (12.1%) continued ICI without subsequent TKI use, whereas 20 (5.5%) eventually discontinued ICI and received subsequent TKI. In total, 108 patients (29.7%) switched to TKIs at the point of progression on ICI, whereas another 27 patients (7.4%) were classified as receiving other anticancer therapies post‐progression on ICI, including external beam radiotherapy in 15 patients and trans‐arterial chemoembolization (TACE) in 6. Of the 364 study patients, 9 received a different ICI or ICI‐containing regime post‐progression, all of whom also received a TKI at the point of progression.

Baseline characteristics of the overall study population and patients according to the receipt of any post‐progression anticancer treatment are summarised in Table 1. Receipt of post‐ICI progression therapy of any kind was more common in patients with better ECOG‐PS at baseline and at disease progression (*p* = .0005 and *p* < .0001 respectively), better baseline ALBI grade (*p* = .0073), and in those who had received liver resection prior to ICI (37.7% vs. 24.2%, *p* = .0061). No other baseline features were found to be associated with the receipt of post‐progression treatments, including age, presence of cirrhosis, Child‐Pugh class, BCLC stage and ICI treatment line. ALBI grade variation and ALBI grade at disease progression were not associated with the receipt of post‐progression active therapy. Median PFS from ICI commencement for the whole study population was 3.2 months and not significantly different when patients were categorised according to receipt post‐progression therapy of any type (3.5 months in those who received further therapy vs. 2.8 months in those who did not, *p* = .1146).

**TABLE 1:** Study patient demographics and clinical characteristics

Baseline characteristics (except where specified at PD)	Overall *n* = 364 (%)	Patients receiving post‐progression therapy *n* = 199 (54.7%)	Patients not receiving post‐progression therapy *n* = 165 (45.3%)	Chi‐square
Age (years: median (range))	66 (25–86)	66 (25–86)	67 (39–86)	*p* = .3721
<70	247 (67.9)	139 (69.8)	108 (65.6)	
≥70	117 (32.1)	60 (30.2)	57 (34.5)	
Gender				*p* = .9052
Male	290 (79.7)	159 (79.9)	131 (79.4)	
Female	74 (20.3)	40 (20.1)	34 (20.6)	
ECOG‐PS (baseline)				***p* = .0005**
0	203 (55.8)	124 (62.3)	79 (47.9)	
1	149 (40.9)	74 (37.2)	75 (45.5)	
≥2	12 (3.3)	1 (0.5)	11 (6.7)	
ECOG‐PS at PD				***p* < .0001**
0	108 (31.6)	72 (38.3)	36 (23.4)	
1	145 (42.4)	86 (45.7)	59 (38.3)	
≥2	89 (26.0)	30 (16.0)	59 (38.2)	
Missing^a^	22	11	11	
Cirrhosis				*p* = .5441
Absent	114 (31.3)	65 (32.7)	49 (29.7)	
Present	250 (68.7)	134 (67.3)	116 (70.3)	
Viral or non‐viral HCC				*p* = .6973
Non‐viral HCC	157 (43.1)	84 (42.2)	73 (44.2)	
HBV and/or HCV infection	207 (56.9)	115 (57.8)	92 (55.8)	
Child‐Pugh Class				p = .1219
A	268 (73.6)	153 (76.9)	115 (69.7)	
B	96 (26.4)	46 (23.1)	50 (30.3)	
BCLC stage				*p* = .1140
A	13 (3.6)	5 (2.5)	8 (4.8)	
B	48 (13.2)	32 (16.1)	16 (9.7)	
C	303 (83.2)	162 (81.4)	141 (85.5)	
AFP (ng/ml)				*p* = .3260
<400	203 (57.8)	110 (55.6)	93 (60.8)	
>400	148 (42.2)	88 (44.4)	60 (39.2)	
Missing^a^	13	1	12	
ALBI grade (baseline)				***p* = .0073**
1	102 (28.0)	61 (30.7)	41 (24.8)	
2	171 (47.0)	79 (39.7)	92 (55.8)	
3	91 (25.0)	59 (29.6)	32 (19.4)	
ALBI grade at PD				*p* = .7132
1	57 (18.4)	35 (20.0)	22 (16.4)	
2	129 (41.7)	71 (40.6)	58 (43.3)	
3	123 (39.8)	69 (39.4)	54 (40.3)	
Missing^a^	55	24	31	
ALBI grade variation				*p* = .1752
Deterioration	75 (24.3)	36 (20.6)	39 (29.1)	
Unchanged	224 (72.5)	132 (75.4)	92 (68.7)	
Improvement	10 (3.2)	7 (4.0)	3 (2.2)	
Missing^a^	55	24	31	
Prior treatment for HCC				
Resection	115 (31.6)	75 (37.7)	40 (24.2)	***p* = .0061**
Trans‐arterial chemoembolization	182 (50.0)	105 (52.8)	77 (46.7)	*p* = .2474
Sorafenib	193 (53.0)	99 (49.7)	94 (57.0)	*p* = .1700
Treatment line				*p* = .3546
First systemic line	160 (44.0)	93 (46.7)	67 (40.6)	
Second systemic line	155 (42.6)	78 (39.2)	77 (46.7)	
Beyond the second systemic line	49 (13.5)	28 (14.1)	21 (12.7)	
ICI regime				***p* = .0005**
Anti‐PD‐(L)1 monotherapy	292 (80.2)	175 (87.9)	117 (70.9)	
Anti‐PD‐(L)1 + CTLA‐4 combination	23 (6.3)	9 (4.5)	14 (8.5)	
Anti‐PD‐(L)1 + TKI combination	32 (8.8)	7 (3.5)	25 (15.1)	
Atezolizumab/bevacizumab	17 (4.7)	8 (4.0)	9 (5.5)	
				Mann–Whitney
PFS from ICI commencement (months (IQR))	3.2 (1.9–7.0)	3.5 (2.1–7.2)	2.8 (1.8–7.0)	*p* = .1146

*Note*: Variables are compared between patients receiving post‐progression therapy of any kind and those who do not with chi‐square tests.

Abbreviations: ALBI, albumin‐bilirubin; BCLC, Barcelona Clinic Liver Cancer; CTLA‐4, cytotoxic T‐lymphocyte‐associated protein‐4; ECOG‐PS, Eastern Cooperative Oncology Group performance status; HCC, hepatocellular carcinoma; ICI, immune checkpoint inhibitor; PD‐(L)1, programmed cell death ligand‐1; PD, progressive disease; PFS, progression‐free survival; TKI, tyrosine kinase inhibitor.

Bold values are set for *p* < .05.

^a^ Missing values were excluded from the computation of proportions and *p*‐values.

### 3.2 Continuation of immune checkpoint inhibitor post‐progression and treatment switching to tyrosine kinase inhibitor are both associated with improved post‐progression survival

With a median post‐progression follow‐up of 22.8 months (95% CI: 19.4–26.5), the median PPS for the study population was 5.3 months (95% CI: 4.4–6.9; 271 events) (Figure S1A). Receipt of post‐progression treatment was significantly associated with a prolonged PPS (10.3 months vs. 1.9 months, *p* < .0001; Figure S1B).

**FIGURE 2:**
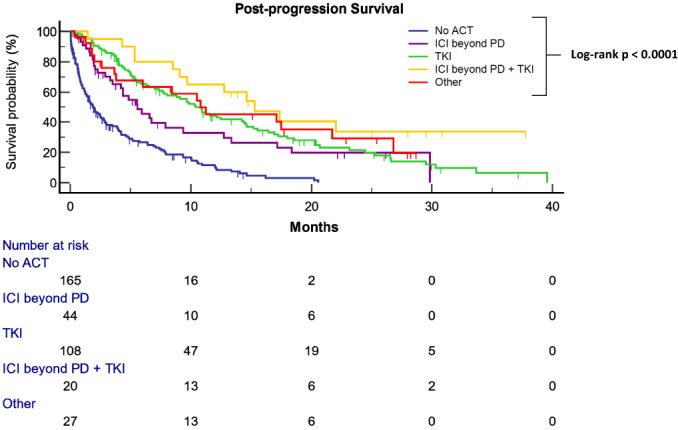
Kaplan–Meier curves of post‐progression survival (PPS) in hepatocellular carcinoma patients treated with immune checkpoint inhibitor (ICI) according to treatment strategy. Patients who did not receive post‐progression anticancer therapy (no ACT): 1.9 months (95% CI: 1.3–2.7, 132 events), patients who received ICIs beyond PD only (ICI beyond PD): 5.6 months (95% CI: 3.5–9.4, 31 events), patients who received post‐PD tyrosine kinase inhibitors (TKIs) only (TKI): 10.4 months (95% CI: 7.7–14.4, 79 events), patients who received ICIs beyond PD followed by TKIs (ICI beyond PD + TKI): 15.3 months (95% CI: 8.5–22.0, 12 events), patients who received other post‐PD anticancer therapies (other): 10.8 months (95% CI: 3.7–21.7, 17 events).

Figure 2 describes PPS estimates for different post‐progression therapeutic approaches as categorised into the five previously described groups: permanent cessation of anticancer therapy, ICI discontinuation and switch to TKI, continuation of ICI beyond PD with and without subsequent TKI switch, and other anticancer therapy. All four active treatment approaches were associated with an improved PPS compared to no anticancer therapy (HRs from univariable analyses are available in Table 3). Figure S2 describes PPS estimates for patients receiving ICI‐based regimes as first‐line therapy, categorised into the same groups.

### 3.3 New vascular invasion and intra‐hepatic tumour growth predict poorer post‐progression survival at disease progression

Among the 277 patients with available information, the radiological patterns of progression were 178 (64.3%) IHG, 63 (22.7%) NIH, 119 (43.0%) EHG, 72 (26.0%) NEH and 28 (10.1%) nVI. Progression pattern overlaps are detailed in Table S2.

Clinical and radiological features at disease progression may influence clinician behaviour as well as survival outcome. We therefore verified the relationship between these features and treatment strategy at PD on ICI: ECOG‐PS and ALBI grade at PD were found to be significant determinants of treatment strategy (Table 2).

**TABLE 2:** Distribution of patterns of disease progression and clinical features according to treatment strategy at PD on ICI

Characteristic	No post‐progression anticancer therapy	ICI beyond PD without subsequent TKI	Post‐PD TKI (without ICI beyond PD)	ICI beyond PD with subsequent TKI	Other post‐PD therapies	
IHG						*p* = .2216
No	32 (29.1)	16 (42.1)	32 (36.0)	7 (41.2)	12 (52.2)	
Yes	78 (70.9)	22 (57.8)	57 (64.0)	10 (58.8)	11 (47.8)	
Missing^a^	55	6	19	3	4	
NIH						*p* = .1167
No	93 (84.5)	30 (78.9)	61 (68.5)	13 (76.5)	17 (73.9)	
Yes	17 (15.5)	8 (21.1)	28 (31.5)	4 (23.5)	6 (26.1)	
Missing^a^	55	6	19	3	4	
EHG						*p* = .3484
No	66 (60.0)	16 (42.1)	54 (60.7)	9 (52.9)	13 (56.5)	
Yes	44 (40.0)	22 (57.9)	35 (39.3)	8 (47.1)	10 (43.5)	
Missing^a^	55	6	19	3	4	
NEH						*p* = .4027
No	86 (78.2)	29 (76.3)	61 (68.5)	14 (82.4)	15 (65.2)	
Yes	24 (21.8)	9 (23.7)	28 (31.5)	3 (17.6)	8 (34.8)	
Missing^a^	55	6	19	3	4	
nVI						*p* = .4631
No	96 (87.3)	34 (89.5)	81 (91.0)	15 (88.2)	23 (100)	
Yes	14 (12.7)	4 (10.5)	8 (9.0)	2 (11.8)	—	
Missing^a^	55	6	19	3	4	
Extra‐hepatic progression^b^						*p* = .3321
No	53 (48.2)	11 (28.9)	40 (44.9)	8 (47.1)	9 (39.1)	
Yes	57 (51.8)	27 (71.1)	49 (55.1)	9 (52.9)	14 (60.9)	
Missing^a^	55	6	19	3	4	
Pattern of progression						*p* = .1685
Without new lesions	63 (57.3)	21 (55.3)	36 (40.4)	9 (52.9)	10 (43.5)	
With new lesions	47 (42.7)	17 (44.7)	53 (59.6)	8 (47.1)	13 (56.5)	
Missing^a^	55	6	19	3	4	
ECOG‐PS at PD						***p* < .0001**
0	36 (23.4)	10 (25.0)	43 (41.7)	8 (42.1)	11 (42.3)	
1	59 (38.3)	18 (45.0)	50 (48.5)	10 (52.6)	8 (30.8)	
≥2	59 (38.3)	12 (30.0)	10 (9.7)	1 (5.3)	7 (26.9)	
Missing^a^	11	4	5	1	1	
ALBI grade at PD						***p* = .0460**
1	22 (16.4)	8 (20.0)	15 (16.3)	8 (44.4)	4 (16.0)	
2	58 (43.3)	16 (40.0)	41 (44.6)	8 (44.4)	6 (24.0)	
3	54 (40.3)	16 (40.0)	36 (39.1)	2 (11.1)	15 (60.0)	
Missing^a^	31	4	16	2	2	
ALBI grade variation						*p* = .1941
Deterioration	39 (29.1)	10 (25.0)	20 (21.7)	4 (22.2)	2 (8.0)	
Unchanged	92 (68.7)	28 (70.0)	69 (75.0)	12 (66.7)	23 (92.0)	
Improvement	4	2	3 (3.3)	2 (11.1)	—	
Missing^a^	31	4	16	2	2	
ICI treatment line						*p* = .5158
First systemic line	67 (40.6)	22 (50.0)	46 (42.6)	10 (50.0)	15 (55.6)	
Non‐first systemic line	98 (59.4)	22 (50.0)	62 (57.4)	10 (50.0)	12 (44.4)	

Abbreviations: ALBI, albumin‐bilirubin; ECOG‐PS, Eastern Cooperative Oncology Group performance status; EHG, extrahepatic growth; ICI, immune checkpoint inhibitor; IHG, intrahepatic growth; NEH, new extrahepatic lesion(s); NIH, new intrahepatic lesion(s); nVI, new vascular invasion; PD, progressive disease; TKI, tyrosine kinase inhibitor.

Bold values are set for *p* < .05.

^a^ Missing values were excluded from the computation of proportions.

^b^ nVI included within intrahepatic disease progression.

Table 3 summarises the univariable and multivariable analyses of PPS. Among radiological patterns of progression, IHG (HR 1.64 [95% CI: 1.21–2.22]; *p* = .0013) and nVI (HR 2.15 [95% CI: 1.38–3.35]; *p* = .0007) were significantly associated with a poorer PPS in the univariable analysis. After adjusting for all progression patterns, ECOG‐PS at disease progression, ALBI score at disease progression, treatment strategy and treatment line of ICI in a multivariable analysis, nVI remained significantly associated with poorer prognosis (HR 2.16 [95% CI: 1.35–3.46]; *p* = .0012), while IHG was no longer significantly associated with PPS. ECOG‐PS score of ≥2 was significantly associated with a shorter PPS in univariable (HR 3.17 [95% CI: 2.27–4.23]; *p* < .0001) and multivariable analyses (HR 2.04 [95% CI: 1.31–3.17]; *p* = .0015). All categories of post‐progression therapy were associated with an improved PPS compared to no anticancer therapy in both the univariable and multivariable analyses. Figure S3 illustrates a multivariable survival probability plot according to the presence of each given pattern of progression in the whole study population, providing a graphical representation of the increased risk of death for patients experiencing nVI, regardless of disease progression to other sites.

**TABLE 3:** Univariable and multivariable analyses of post‐progression survival

Variable	Post‐progression survival (PPS)			
	No. of patients	Univariable analysis HR (95% CI); *p*‐value	No. of patients	Multivariable analysis HR (95% CI); *p*‐value
Post‐progression therapy	364		265	
No post‐progression anticancer therapy		1		1
ICI beyond PD without subsequent TKI		0.39 (0.26–0.58); ***p* < .0001**		0.52 (0.32–0.84); ***p* = .0075**
Post‐PD TKI (without ICI beyond PD)		0.29 (0.22–0.39); ***p* < .0001**		0.38 (0.25–0.56); ***p* < .0001**
ICI beyond PD with subsequent TKI		0.17 (0.09–0.32); ***p* < .0001**		0.24 (0.12–0.49); ***p* = .0001**
Other post‐PD therapies		0.26 (0.15–0.43); ***p* < .0001**		0.41 (0.22–0.73); ***p* = .0031**
IHG				
Yes versus No	277	1.64 (1.21–2.22); ***p* = .0013**		1.25 (0.88–1.79); *p* = .2088
NIH				
Yes versus No	*277*	0.80 (0.57–1.13); *p* = .2116		1.08 (0.74–1.57); *p* = .6631
EHG				
Yes versus No	277	0.98 (0.74–1.31); *p* = .9245		1.15 (0.85–1.55); *p* = .3377
NEH				
Yes versus No	*277*	1.05 (0.76–1.43); *p* = .7594		1.07 (0.76–1.50); *p* = .7077
nVI				
Yes versus No	277	2.15 (1.38–3.35); ***p* = .0007**		2.16 (1.35–3.46); ***p* = .0012**
ALBI grade at disease progression	309			
1		1		1
2		1.38 (0.94–2.01); *p* = .0940		0.97 (0.62–1.50); *p* = .8943
3		1.51 (1.04–2.21); ***p* = .0334**		1.16 (0.73–1.84); *p* = .5087
ECOG‐PS at disease progression	342			
0		1		1
1		1.30 (0.96–1.76); *p* = .0940		1.07 (0.76–1.50); *p* = .6958
≥2		3.17 (2.27–4.43); ***p* < .0001**		2.04 (1.31–3.17); ***p* = .0015**
ICIs treatment line				
First versus non‐first	364	1.03 (0.81–1.31); *p* = .8040		0.87 (0.64–1.16); *p* = .3407

Abbreviations: ALBI, albumin‐bilirubin; ECOG‐PS, Eastern Cooperative Oncology Group performance status; EHG, extrahepatic growth; HR, hazard ratio; ICI, immune checkpoint inhibitor; IHG, intrahepatic growth; NEH, new extrahepatic lesion(s); NIH, new intrahepatic lesion(s); nVI, new vascular invasion; PD, progressive disease; TKI, tyrosine kinase inhibitor.

Bold values are set for *p* < .05.

**FIGURE 3:**
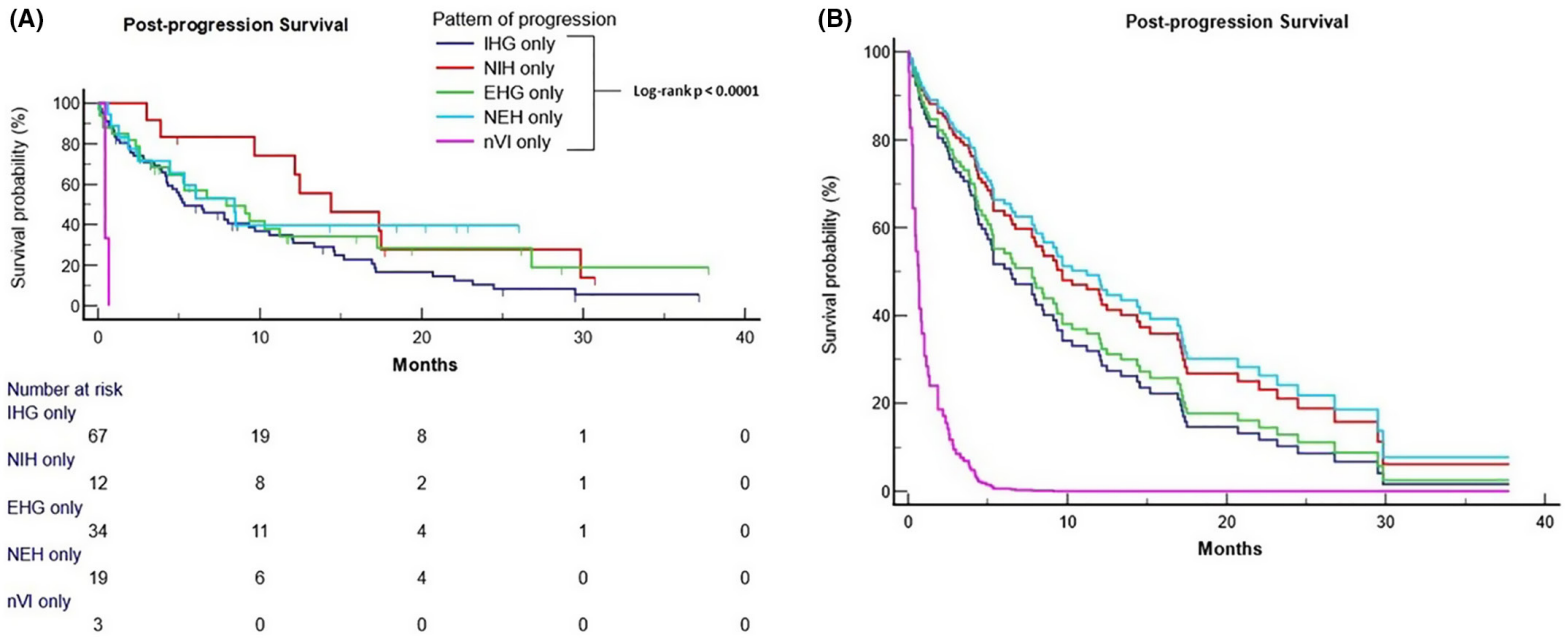
(A) Kaplan–Meier survival estimates for post‐progression survival (PPS) according to the radiological pattern of progression. Intrahepatic growth only: 5.3 months (95% CI: 4.2–9.7; 54 events), new intrahepatic lesion only: 14.4 months (95% CI: 3.8–29.8; 9 events), extrahepatic growth only: 7.9 months (95% CI: 3.3–17.3; 21 events), new extrahepatic lesion only: 8.4 months (95% CI: 2.5–8.5; 10 events), new vascular invasion only: 0.4 months (95% CI: 0.4–0.6; 3 events). (B) Multivariable Cox regression survival probability plot for PPS according to the pattern of progression.

The ancillary analysis included 135 patients with non‐overlapped patterns of disease progression. Figure 3 reports PPS for patients experiencing single (non‐overlapping) patterns of progression, with patients experiencing combined patterns excluded: IHG median PPS 5.3 months, NIH 14.4 months, EHG 7.9 months, NEH 8.4 months and nVI 0.4 months (*p* < .0001). Table S3 summarises the univariable and multivariable analysis of PPS including the pattern of progression as a unique categorical covariate. After adjusting for the type of post‐progression therapy received, ALBI grade at disease progression, ECOG‐PS at disease progression, and ICI treatment line, only nVI was confirmed as independently associated with an increased post‐progression risk of death (HR 6.41 [95% CI: 1.68–24.37]; *p* = .0064).

## 4 DISCUSSION

The systemic therapy armamentarium of HCC now recognises multiple therapeutic options across lines of therapy. Widening therapeutic options pose significant challenges to clinicians treating HCC patients, especially in view of the lack of biomarkers that can facilitate the selection of the most appropriate therapy a priori.^10^, ^16^ In this large, multi‐institutional observational study of 364 patients, largely treated with ICI monotherapy post‐sorafenib failure, we document that in patients who discontinue ICI owing to disease progression the overall prognosis is remarkably heterogeneous. Among patients who are eligible to further therapy, dynamic changes in the radiological distribution of disease identify subsets of patients with diverse PPS.

Interestingly, we show that unlike patients who progress on sorafenib where NEH and nVI are dominant features portending to adverse prognosis,^7^ in the setting of ICI therapy IHG and nVI appear to be associated with worse PPS, with only nVI qualifying as an independent predictor of poorer survival in multi‐variable analyses.

The angiotropic nature of HCC is a characteristic of HCC progression, where both microscopic vascular invasion and macroscopic portal vein spread stand among the key clinicopathological factors predicting an adverse disease course in early^17^, ^18^ and advanced stage disease^19^ respectively. In a landmark study by Iavarone et al. investigating prognostic factors in patients who permanently discontinued sorafenib, macrovascular invasion was confirmed as a key adverse prognostic factor in patients with HCC, emphasising the importance of assessing dynamic changes in tumour burden when re‐assessing patients for further lines of systemic therapy.^20^

Our study portrays a substantial difference in the prognostic value of post‐progression radiological features compared to the sorafenib era, where NEH and nVI led to the subclassification of the BCLC stage based on characteristics evident upon progression on sorafenib.^21^ It should be acknowledged that NEH and nVI were categorised together in previous analyses of post‐progression outcomes following treatment with sorafenib,^7^ as well as ramucirumab^22^: we chose to evaluate these two patterns of progression separately, postulating that they could reflect different underlying tumour biology. It is plausible that differences in the mechanism of action between TKIs and immunotherapy might explain the difference in the prognostic weight of these variables. Immunotherapy is in fact more likely than sorafenib to produce radiologically appreciable disease responses and is capable of leading to long‐term disease stabilisation in up to 20% of patients with HCC.^3^, ^4^ Although patients who respond to immunotherapy tend to be those that benefit most in terms of survival,^23^ evolving experience in the use of PD‐(L)1 inhibitors in indications other than HCC suggests the potential for treatment‐induced benefit even in those patients who fail to achieve a significant radiological response; a finding that does not apply to TKIs.

When evaluating the clinical use of ICI in routine clinical practice, we were able to describe that >50% of patients received subsequent anticancer treatment after progression on ICI‐based therapy: a higher percentage compared to the 23.6% of patients receiving second‐line therapy in previous studies.^24^ In an increasingly complex treatment landscape, our study shows that sequential exposure to multiple agents characterised by disease‐modulating activity in HCC is associated with incremental survival benefits in patients who remain fit for treatment, a consolidated concept from the TKI era in HCC.^25^, ^26^ While no level I evidence exists to document the efficacy of TKI use after ICI, our study provides important hypothesis‐generating evidence to suggest the utility of TKI use after ICI failure, in line with other recently published observational studies^27^ and a recent simulation model of ICI‐TKI sequencing.^28^ It should be noted that 53% of patients in our study had received sorafenib prior to ICI, which no longer reflects the current standard‐of‐care algorithm. Start dates of initial first‐line systemic therapy were unfortunately not available: other prospectively maintained cohorts with these data could provide updated estimates of OS from this point and validate different therapeutic sequencing approaches.

While retrospective interrogation of the reasoning behind therapeutic sequencing carries undeniable selection bias, our study consolidates the role of ECOG‐PS and liver dysfunction as determinants influencing outcomes after the progression of ICI. ECOG‐PS at disease progression was strongly associated with overall receipt of post‐progression therapy (*p* < .0001, Table 1), and treatment strategy (*p* < .0001, Table 2). ALBI grade at ICI commencement (*p* = .007, Table 1) but not at the point of progression (*p* = .71) was associated with receipt of post‐progression therapy of any kind. ALBI grade deterioration from baseline was more prevalent in patients not receiving further therapy compared to those who did (29.1% vs. 20.6%), although without reaching statistical significance (*p* = .18). ALBI grade at PD was however a determinant of specific post‐progression strategy (Table 2, *p* = .046). Why liver function at the commencement of ICI rather than at progression is associated with the overall receipt of further therapy in this study is not clear. Prior therapy is a possible confounding factor herein: sorafenib pre‐treatment was more common in patients not receiving post‐progression therapy (57.0% vs. 49.7%). However, this difference was non‐significant (*p* = .17, Table 1) and treatment line of ICI itself was not associated with receipt of post‐progression therapy (*p* = .35), or therapeutic strategy at PD (*p* = .52, Table 2).

Regarding other potential determinants of receipt of post‐progression therapy, it could be argued that adverse disease biology or intrinsic treatment resistance may preclude eligibility, or influence the clinician decision to treat further. However, PFS from ICI initiation was not significantly lower in patients who did not receive further lines of therapy (median 2.8 months vs. 3.5 months, *p* = .11, Table 1). Patients not receiving post‐progression therapy were more commonly treated with ICI in combination with a second agent than those who did receive further therapy (*p* = .0005). This could reflect reduced availability of other drug classes or greater treatment toxicity. Prior liver resection was more common in patients receiving post‐progression therapy than those who did not (37.7% vs. 24.2%, *p* = .0061). This may reflect a low burden of relapsed disease detected on surveillance following curative‐intent surgery.

In our study, 17.6% of patients who progressed on ICI continued immunotherapy treatment beyond initial evidence of radiological progression, mostly in the context of the growth of existing lesions. The retrospective, non‐randomised nature of our data cannot constitute a platform for practice‐changing recommendations. However, the median PPS of 15.3 months achieved by patients whose change to TKI therapy was deferred beyond initial evidence of progression to ICI is a provocative finding and compares favourably to survival estimates of patients who were treated with immediate switching to TKI. Continuation of ICI beyond the first point of progression is commonplace in clinical practice and often permitted in clinical trials of ICI, recognising the potential for cancer immunotherapy to alter tumour biology beyond strict radiological criteria^29^ and allowing, in other tumour types, deferred switch to potentially less tolerable strategies such as chemotherapy^30^, ^31^ or TKIs.^32^

To our knowledge, our study is the first to report on the clinical use of immunotherapy beyond RECIST progression in HCC and suggests that this strategy should be investigated in a subset of patients with advanced disease, potentially based on the radiographic characteristics of progression. The dissociation between radiological response and PPS in patients treated with ICI highlights the need for further research into modified imaging criteria and alternative immune monitoring biomarkers to identify patients who may derive greater benefit from immunotherapy.^33^

Limitations to this study should also be acknowledged. The retrospective nature of the dataset, although prospectively maintained, limits the ability to draw definitive conclusions about post‐progression outcomes, and the benefit of this approach over immediate switching to other effective therapies remains to be evaluated prospectively. Despite recruitment to the study being multi‐centre, selection bias needs to be considered and clinical practice at non‐participating sites may be different. Prescribing practice (and consequently patient outcomes) may be skewed by differences in treatment availability and reimbursement policies across countries. Pembrolizumab is approved as a second‐line agent in the USA but not in the UK, which should be acknowledged as a potential confounding factor. Tumour imaging was also reviewed locally rather than centrally. Efforts were made to reduce selection bias through the employment of multivariable analyses, but the potential remains for other variables to confound associations with PPS.

Despite these potential limitations, this study gives a contemporary portrait of how immunotherapy has integrated the management of advanced HCC. In spite of the heterogeneity in prognosis, partly dictated by the diverse radiological patterns of disease progression, more than 50% of patients with HCC who experience PD in ICI receive subsequent therapy. Prospective research efforts should validate the impact of radiological patterns of disease progression and continuation of ICI post‐radiological PD in patients with advanced HCC in order to optimise treatment sequencing in immunotherapy‐resistant disease.

## FUNDING INFORMATION

A. D'Alessio is supported by grant funding from the European Association for the Study of the Liver (Andrew Burroughs Fellowship). D. Bettinger is supported by the Berta‐Ottenstein Programme, Faculty of Medicine, University of Freiburg. A. Cortellini is supported by grant funding from the NIHR Imperial College BRC. D. J. Pinato is supported by grant funding from the Wellcome Trust Strategic Fund (PS3416).

## CONFLICT OF INTEREST

M. Pinter is an investigator for Bayer, BMS, Lilly and Roche; he received speaker honoraria from Bayer, BMS, Eisai, Lilly and MSD; he is a consultant for Bayer, BMS, Eisai, Ipsen, Lilly, MSD and Roche; he received travel support from Bayer and BMS. B. Scheiner received travel support from Gilead, Ipsen and AbbVie. T.U. Marron serves on advisory and/or data safety boards for Rockefeller University, Regeneron, BMS, Boehringer Ingelheim, Atara, AstraZeneca, Genentech, Celldex, Surface, NGMbio, DBV Technologies and receives research funding from Regeneron, Bristol‐Myers Squibb, Merck, Boehringer Ingelheim. T. Jun owns stock and is employed by Sema4. D. Bettinger has received lecture and speaker fees from Bayer Healthcare, the Falk Foundation Germany and consulting fees from Boston Scientific. T. Pressiani received consulting fees from Bayer; and institutional research funding from Bayer, Lilly and Roche. N. Personeni received consulting fees from Amgen, Merck Serono and Servier; lectures fees from AbbVie, Gilead, Lilly and Sanofi; travel expenses from Amgen, ArQule and institutional research funding from Basilea, Merck Serono and Servier. L. Rimassa received consulting fees from Amgen, ArQule, AstraZeneca, Basilea, Bayer, BMS, Celgene, Eisai, Exelixis, Genenta, Hengrui, Incyte, Ipsen, IQVIA, Lilly, MSD, Nerviano Medical Sciences, Roche, Sanofi, Servier, Taiho Oncology, Zymeworks; lecture fees from AbbVie, Amgen, Bayer, Eisai, Gilead, Incyte, Ipsen, Lilly, Merck Serono, Roche, Sanofi; travel expenses from Ipsen and institutional research funding from Agios, ARMO BioSciences, AstraZeneca, BeiGene, Eisai, Exelixis, Fibrogen, Incyte, Ipsen, Lilly, MSD, Nerviano Medical Sciences, Roche, Zymeworks. A. Vogel reports honoraria for speaker, consultancy and advisory role from Roche, AstraZeneca, EISAI, Bayer, Merck, Bristol Myers Squibb, Merck Sharp & Dohme, Incyte, PierreFabre, Ipsen and Sanofi. P. R. Galle reports a consulting or advisory role and received honoraria from AdaptImmune, AstraZeneca, Bayer, Bristol Myers Squibb, Eisai, Ipsen, Lilly, Merck Sharp & Dohme, Roche and Sirtex; has been on a speakers bureau for AstraZeneca, Bayer, Bristol Myers Squibb, Eisai, Ipsen, Lilly, Merck Sharp & Dohme, Roche and Sirtex; has received research funding from Bayer and Roche; has provided expert testimony for Lilly and has received travel or accommodation expenses from AstraZeneca, Bayer, Bristol Myers Squibb, Eisai, Ipsen, Lilly, and Roche. A. Cortellini received grant consultancies and speaker fees from AstraZeneca, BMS, Roche, MSD, Novartis, Eisai outside the submitted work. D. J. Pinato received lecture fees from ViiV Healthcare and Bayer Healthcare and travel expenses from BMS and Bayer Healthcare; consulting fees for Mina Therapeutics, EISAI, Roche and Astra Zeneca; received research funding (to institution) from MSD and BMS. All remaining authors have declared no conflict of interest. The authors have no other relevant affiliations or financial involvement with any organization or entity with a financial interest in or financial conflict with the subject matter or materials discussed in the manuscript apart from those disclosed. No writing assistance was utilised in the production of this manuscript.

## Supporting information


**Appendix S1:** Supporting Information
