# Supplementary material for: Progression patterns and therapeutic sequencing following immune checkpoint inhibition for hepatocellular carcinoma: An international observational study
Source: Liver Int. 2023 Jan 13;43(3):695–707. doi: 10.1111/liv.15502 (PMC10947007; doi:10.1111/liv.15502)

Supplementary Material

**Contents**

**Supplementary Table 1 Page 2**

Participating institutions.

**Supplementary Table 2** **Page 3**

Detailed description of disease progression patterns overlaps.

**Supplementary Table 3**   **Page 4**

Multivariable and univariable analysis of PPS including patients who experienced non-overlapping patterns of disease of progression.

**Supplementary Figure 1 Page 5**

Kaplan-Meier survival estimates. **a)** PPS for the overall study population. **b)** PPS according to receipt of any post-progression anticancer therapy.

**Supplementary Figure 2 Page 6**

Kaplan-Meier curves of PPS in patients receiving ICI therapy as first line according to treatment strategy at PD.

**Supplementary Figure 3 Page 7**

Cox regression survival probability plot for PPS according to presence of a given radiologic pattern of progression.

**Supplementary Table 1.** Participating institutions.

| **Region,** Institution | **Patients (%)** |
| --- | --- |
| **Europe**  IRCCS Humanitas Research Hospital, Italy  Imperial College London, United Kingdom  Freiburg University, Germany  University of Mainz, Germany  Hannover Medical School, Germany  Medical University Vienna, Austria | 157 (43.1)  82 (22.5)  9 (2.5)  6 (1.6)  16 (4.4)  13 (3.6)  31 (8.5) |
| **North America**  Mount Sinai. New York. USA  Kansas University Medical Center. Kansas. USA  Weill Cornell Medical Center. New York. USA  University of Chicago. Illinois. USA  East Carolina Medical Centre. North Carolina. USA | 126 (34.6)  60 (16.5)  20 (5.5)  19 (5.2)  20 (5.5)  7 (1.9) |
| **Asia**  Taipei Veterans General Hospital. Taiwan  Kindai University. Osaka. Japan | 81 (22.3)  33 (9.1)  48 (13.2) |

|  | **IHG** | | **NIH** | | **EHG** | | **NEH** | | **nVI** | |
| --- | --- | --- | --- | --- | --- | --- | --- | --- | --- | --- |
|  | **No (%)** | **Yes (%)** | **No (%)** | **Yes (%)** | **No (%)** | **Yes (%)** | **No (%)** | **Yes (%)** | **No (%)** | **Yes (%)** |
| **IHG**  No  Yes | **-** | **-** | 77 (36.0)  137 (64.0) | 22 (34.9)  41 (65.1) | 42 (26.6)  116 (73.4) | 57 (47.9)  62 (52.1) | 60 (29.3)  145 (70.7) | 39 (54.2)  33 (45.8) | 95 (38.2)  154 (61.8) | 4 (14.3)  24 (86.7) |
| **NIH**  No  Yes | 77 (77.8)  22 (22.2) | 137 (77.0)  41 (23.0) | **-** | **-** | 114 (72.2)  44 (27.4) | 100 (84.0)  19 (16.0) | 154 (75.1)  51 (24.9) | 60 (83.3)  12 (16.7) | 194 (77.9)  55 (22.1) | 20 (71.4)  8 (28.6) |
| **EHG**  No  Yes | 42 (42.4)  57 (57.6) | 116 (65.2)  62 (34.8) | 114 (53.3)  100 (46.7) | 44 (69.8)  19 (30.2) | **-** | **-** | 121 (59.0)  84 (41.0) | 37 (51.4)  35 (48.6) | 141 (56.6)  108 (43.4) | 17 (60.7)  11 (39.3) |
| **NEH**  No  Yes | 60 (60.6)  39 (39.4) | 145 (81.5)  33 (18.5) | 154 (72.0)  60 (28.0) | 51 (81.0)  12 (19.0) | 121 (76.6)  37 (23.4) | 84 (70.6)  35 (29.4) | **-** | **-** | 182 (73.1)  67 (26.9) | 23 (82.1)  5 (17.9) |
| **nVI**  No  Yes | 95 (96.0)  4 (4.0) | 154 (86.5)  24 (13.5) | 194 (90.7)  20 (9.3) | 55 (87.3)  8 (12.7) | 141 (89.2)  17 (10.8) | 108 (90.8)  11 (9.2) | 182 (88.8)  23 (11.2) | 67 (93.1)  5 (6.9) | **-** | **-** |

**Supplementary Table 2.** Description of radiologic pattern of progression overlap.

**Supplementary Table 3.** Multivariable and univariable analysis of PPS including patients who experienced non-overlapping patterns of disease of progression (used as categorical covariate).

| ***Variable*** | ***Post progression survival (PPS)*** | | | |
| --- | --- | --- | --- | --- |
|  | ***N° of patients*** | **Univariable analysis**  ***HR (95% CI); p-value*** | ***N° of patients*** | **Multivariable analysis**  ***HR (95% CI); p-value*** |
| **Pattern of progression**  IHG  NIH  EHG  NEH  nVI | 135 | 1  0.51 (0.25-1.05); p = 0.0694  0.74 (0.45-1.23); p = 0.2553  0.59 (0.30-1.17); p = 0.1368  11.34 (3.09-41.63); **p = 0.0002** | 126 | 1  1.01 (0.44-2.29); p = 0.9731  1.22 (0.67-2.21); p = 0.5002  0.71 (0.34-1.45); p = 0.3483  6.41 (1.68-24.37); **p = 0.0064** |
| **Post-progression therapy**  No post-progression anticancer therapy  ICI beyond PD without subsequent TKI  Post PD TKI (without ICI beyond PD)  ICI beyond PD with subsequent TKI  Other post PD therapies | 364 | 1  0.39 (0.26-0.58); **p < 0.0001**  0.29 (0.22-0.39); **p < 0.0001**  0.17 (0.09-0.32); **p < 0.0001**  0.26 (0.15-0.43); **p < 0.0001** |  | 1  0.33 (0.14-0.79); **p = 0.0121**  0.27 (0.15-0.51); **p < 0.0001**  0.16 (0.06-0.42); **p = 0.0002**  0.19 (0.08-0.47); **p = 0.0004** |
| **ALBI grade at disease progression**  1  2  3 | 309 | 1  1.38 (0.94-2.01); p = 0.0940  1.51 (1.04-2.21); **p = 0.0334** |  | 1  1.02 (0.55-1.88); p = 0.9394  1.24 (0.66-2.33); p = 0.4841 |
| **ECOG-PS at disease progression**  0  1  ≥ 2 | 342 | 1  1.30 (0.96-1.76); p = 0.0940  3.17 (2.27-4.43); **p < 0.0001** |  | 1  1.43 (0.85-2.39); p = 0.1707  1.69 (0.83-3.42); p = 0.1436 |
| **ICI treatment line**  First vs Non-first | 364 | 1.03 (0.81-1.31); p = 0.8040 |  | 0.71 (0.46-1.12); p = 0.1353 |

**Supplementary Figure 1.** Kaplan-Meier survival estimates. **a)** PPS for the overall study population; median 5.3 months (95%CI: 4.4 – 6.9; 271 events). **b)** PPS of patients who received any post-progression anticancer therapy 10.3 months (95%CI: 7.9 – 12.7; 139 events) vs. patients who did not receive following treatments 1.9 months (95%CI: 1.3 – 2.7; 132 events).

**Supplementary Figure 1.a.**


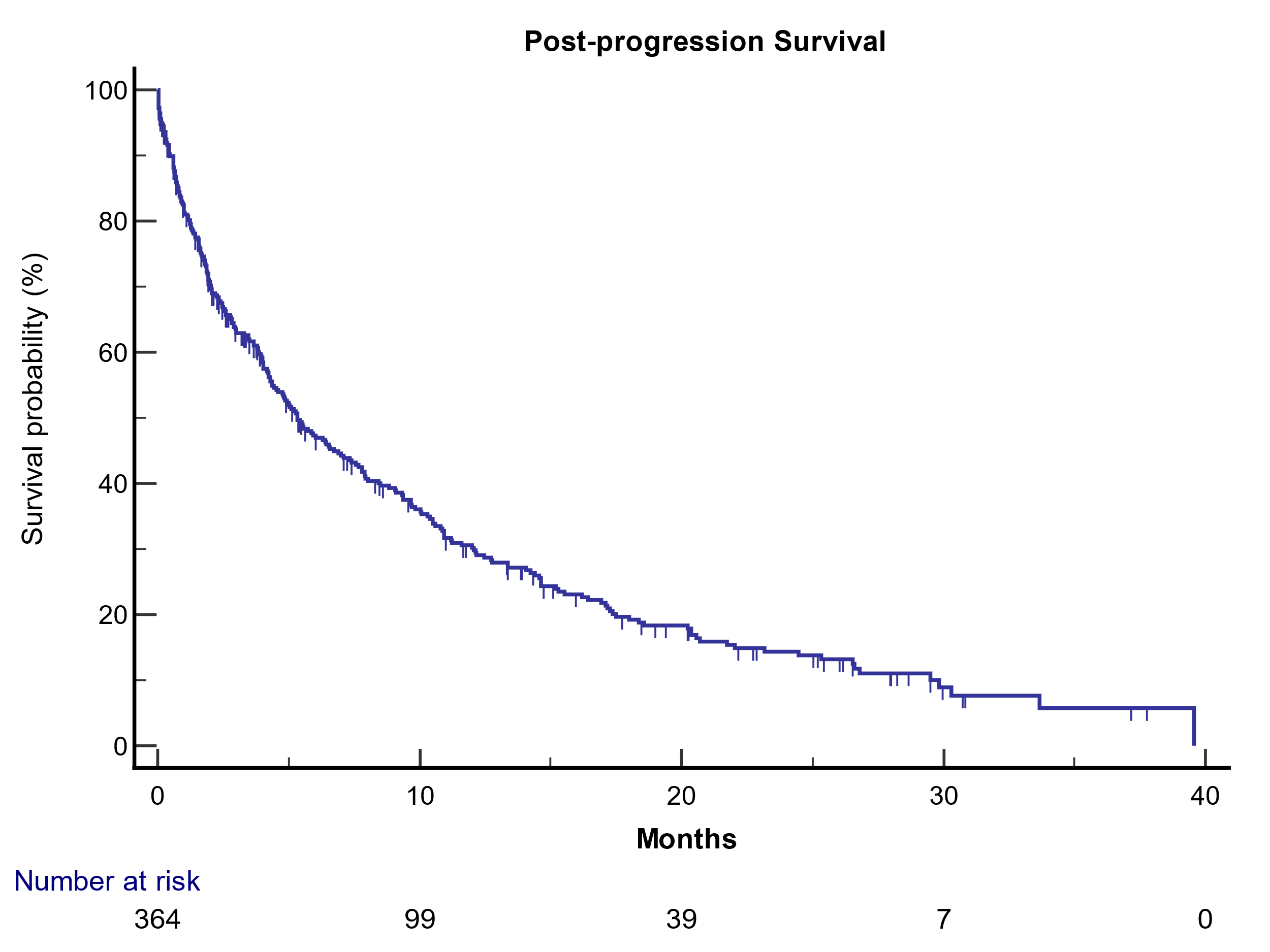


**Supplementary Figure 1.b.**


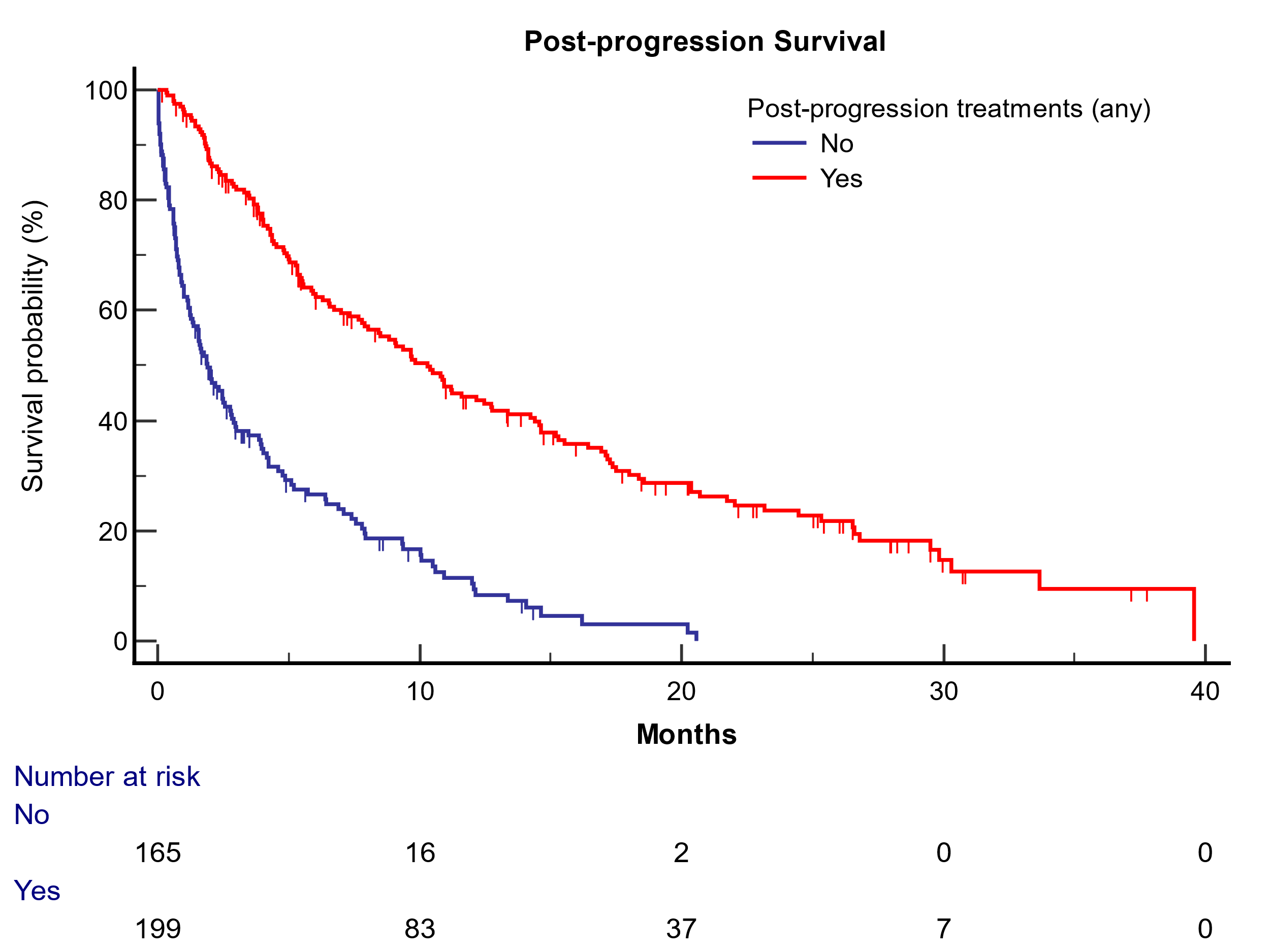


HR=0.23 (95%CI: 0.17-0.31)

**Log-rank p < 0.0001**

**Supplementary Figure 2.** Kaplan-Meier curves of PPS in HCC patients receiving ICI therapy as first line.

Patients who did not receive post progression anticancer therapy (No ACT): 1.3 months (95%CI: 0.8-3.5, 48 events)

Patients who received ICI beyond PD only (ICI beyond PD): 5.6 months (95%CI: 1.9-18.4, 15 events)

Patients who received post PD TKIs only (TKI): 10.9 months (95%CI: 5.0-14.6, 35 events)

Patients who received ICI beyond PD followed by TKIs (ICI beyond PD + TKI): 9.7 months (95%CI: 1.4-22.0, 7 events)

Patients who received other post PD anticancer therapies (Other): 17.5 months (95%CI: 2.0-26.8, 9 events)


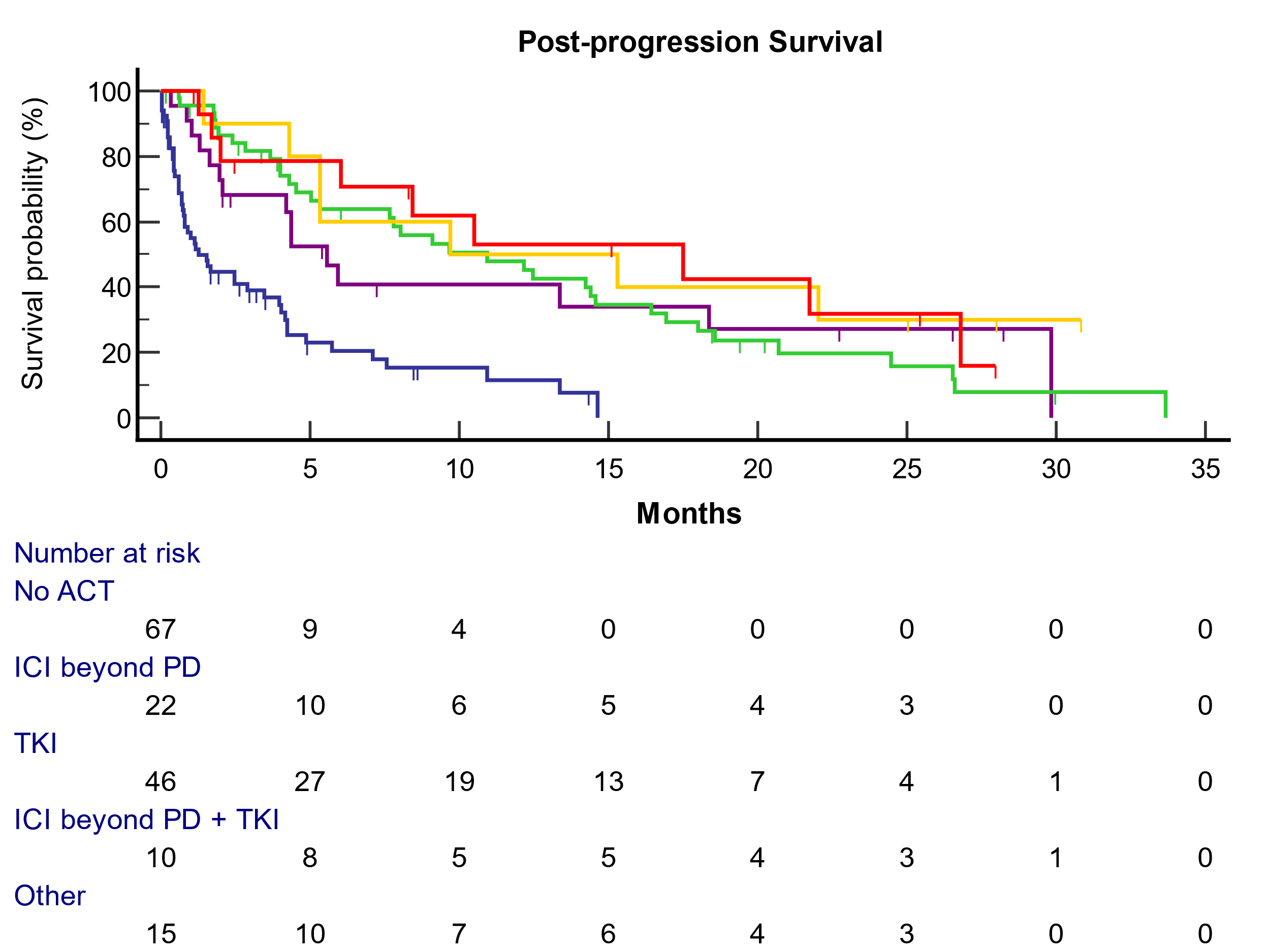


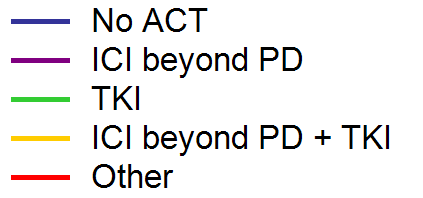


**Log-rank p < 0.0001**

**Supplementary Figure 3.** Cox regression survival probability plot for PPS according to presence of a given radiologic pattern of progression (whole study population). Individual participants typically progress with multiple patterns. Each curve was obtained from separate multivariable models and superimposed, incorporating ECOG-PS at disease progression (0-1 vs ≥ 2), ICI treatment line (1st vs non-1st), ICI beyond PD and post-progression TKIs as adjusting factors.


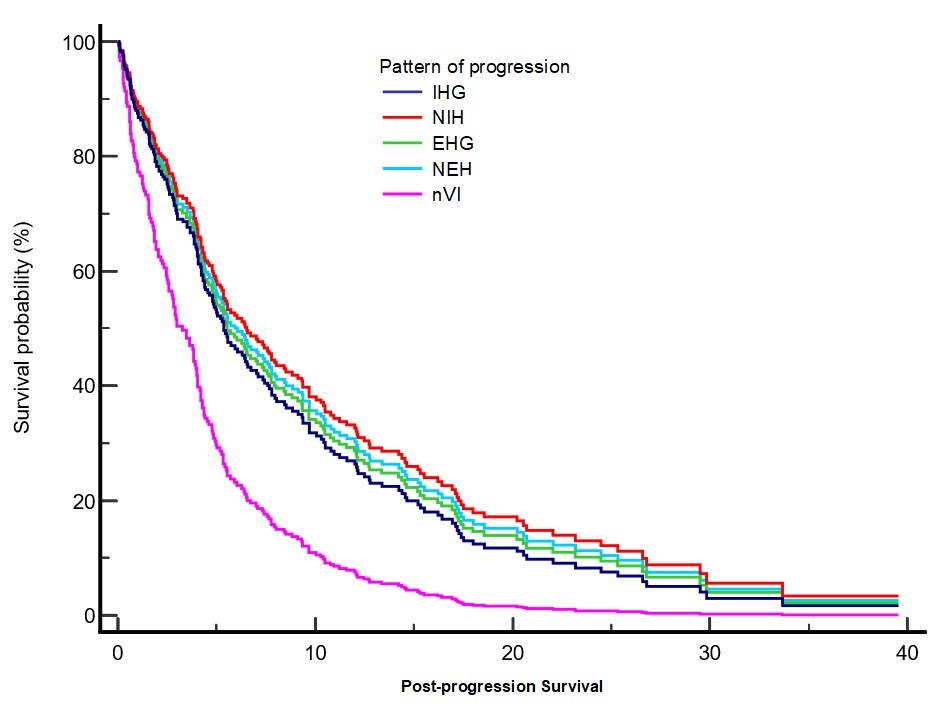

Supplement: Supplementary file 1 — Appendix S1: Supporting Information [file LIV-43-695-s001.docx]
